# Risk factors for recurrent disease in small papillary thyroid cancers – a Swedish register-based study

**DOI:** 10.1007/s00423-023-02905-5

**Published:** 2023-04-26

**Authors:** Haytham Bayadsi, Carolina Nylén, Maria Sandström, Jakob Angelsten, Malin Sund, Joakim Hennings

**Affiliations:** 1 Department of Surgical and Perioperative Sciences/Surgery, Umeå University, Umeå, Sweden; 2 Department of Molecular Medicine and Surgery, Karolinska Institute, Stockholm, Sweden; 3 Department of Radiation Sciences/Oncology, Umeå University, Umeå, Sweden; 4 Department of Surgery, University of Helsinki and Helsinki University Hospital, Helsinki, Finland

**Keywords:** Papillary thyroid cancer (PTC), small PTC, recurrence, lateral lymph node metastasis (N1b), risk factors

## Abstract

**Aims:**

To study the correlation between clinicopathological risk factors and the risk for intervention-requiring cancer recurrence in patients with small papillary thyroid cancers (sPTCs).

**Materials and methods:**

Records for 397 patients with sPTC (T1 ≤ 20mm) were obtained from the Scandinavian Quality Register for Thyroid, Parathyroid and Adrenal Surgery (SQRTPA) between 2010 and 2016. Follow-up time was at least 5 years. Data regarding intervention-requiring cancer recurrence were obtained from patient medical records and analysed regarding lymph node (LN) status (N0, N1a and N1b) and recurrence.

**Results:**

Age was significantly lower in the N1a and N1b groups compared to N0 (45 vs. 40.5 vs. 49 years, respectively; *p* = 0.002). Tumour size was smaller in the N1a group compared to N1b group (9 vs. 11.8 mm; *p* <0.01). The mean number of metastatic LNs at initial surgery was higher in the N1b compared to N1a group (6.6 vs. 3; *p* = 0.001), and in the recurrent compared to the non-recurrent group (7 versus 3.9; *p* <0.01). The recurrence rate was higher in the N1b group than the N1a and N0 groups (25% vs. 2.4% vs. 1.4%, respectively; *p* = 0.001).

**Conclusions:**

Lymph node stage N1b at diagnosis, and having five or more metastatic nodes, are strong risk factors for cancer recurrence and decreased disease-free survival in sPTC. The management of patients with sPTC should include thorough lymph node mapping for optimal treatment and individual risk stratification.

## Introduction

Thyroid cancer (TC) is the most common cancer of the endocrine glands. Papillary thyroid cancer (PTC) is the most common subtype of TC, with an increasing incidence worldwide [1–3]. The rising incidence is partly attributed to established risk factors such as ionizing radiation [1, 2]. Improved diagnostics are also an important contributing factor to the increment. These include ultrasound and fine-needle aspiration cytology, which enable the detection of small papillary thyroid cancers (sPTCs, ≤20mm in size) as well as papillary thyroid microcarcinoma (PTMC, ≤10 mm) [2, 3].

Annual incidence in Sweden per 100,000 people is 8 cases for women and 2.4 cases for men, constituting 550-600 new patients per year [2]. Despite the increasing incidence, the prognosis for sPTC is excellent, with a 10-year survival rate of 90% - 95% [2, 3]. Due to the good prognosis, the general approach is towards less aggressive management of these small tumours [4]. Although most sPTCs are considered low risk cancers, some are considerably more aggressive than others with a high recurrence risk [2, 5–8].

PTC often metastasizes to locoregional cervical lymph nodes. Although the finding of locoregional metastases at diagnosis seems to have a little effect on long-term patient survival [9], it is associated with an increased risk of future locoregional recurrence [10, 11]. Depending on the definition, locoregional recurrences have been described in up to 1.2% - 28% in patients with PTC [9, 12]. Locoregional recurrences are not immediately life-threatening, but they present as a stressor to both physicians and patients. The primary treatment of PTC in Sweden, according to current national guidelines, is total thyroidectomy complemented by postoperative radioactive iodine (RAI) and thyroxine suppression therapy (TST) [2, 13]. For PTMC, hemithyroidectomy is considered sufficient, and in some cases active surveillance can be used in order to avoid surgery [2, 7, 9, 14]. Several options for treatment of recurrent disease are available, depending on the type of recurrence. These include surgery, RAI ablation, external beam radiation therapy (EBRT) and other systemic therapies [2, 13].

The suggested risk factors for cancer recurrence are tumour size, multifocality, vascular invasion, extrathyroidal extension (ETE), lymph node (LN) and distant metastasis [6, 11, 15–18]. Further, LN metastasis location, number, size and ratio (involved LN/retrieved LN) and extranodal extension (ENE) at time of diagnosis are also important risk factors for recurrence [19–24]. Previously, age was believed to be a strong predictive factor of death from thyroid cancer complications, but is now more generally considered in conjunction with other variables [2, 25]. Although females have a higher incidence of PTC, and being male is associated with a higher prevalence of advanced-stage thyroid cancer, gender in general is not considered as a risk factor for recurrence in patients with PTC [26–28]. Molecular biomarkers are not yet widely implemented to help differentiate the relatively small number of aggressive cancers from the larger population of more indolent tumours. The BRAF-V600E mutation carries an increased risk in the intermediate and high risk patient group, but cannot be used as the sole discriminator, as up to 60% of PTCs carry the BRAF-V600E mutation [22, 29]. Telomerase reverse transcriptase (TERT) promoter mutation was found to be an independent risk factor for recurrence and mortality in well differentiated thyroid cancers [30], and, in combination with the BRAF-V600E mutation, TERT promoter mutation is associated with an increased risk of structural disease recurrence [22, 31].

The paradigm shift in management of patients with small PTCs towards less aggressive treatment warrants further understanding of the predictors of cancer recurrence. This would reduce unnecessary treatments for indolent thyroid cancers and improve outcomes in patients with clinically more aggressive cancers.

The aim of this study was to investigate the correlation between demographic factors (age, sex, tumour characteristics such as nodal status, tumour diameter, number of metastasized lymph nodes and multifocality) and the risk for intervention-requiring cancer recurrence in the Swedish sPTC population.

## Materials and methods

### Cohort selection

This study is a registry-based retrospective observational cohort study based on a validated and prospectively maintained register—the Scandinavian Quality Register for Thyroid, Parathyroid and Adrenal Surgery (SQRTPA) [32]. The SQRTPA register was established in 2004 and is the world’s first quality register for endocrine surgery. The SQRTPA covers almost 100% of thyroid surgeries in Sweden. It is validated against the national patient register and is one of the few registers to have an internal quality audit that randomly checks the operating centres every year [32]. The register does not, however, provide longitudinal, long-term follow-up data regarding local and distant recurrence. Therefore, access to the patients’ surgical, oncological, and pathology records was also obtained for the present study.

Patients from all regions across Sweden who underwent surgery between January 2010 and December 2016 were included, which allowed a minimum follow-up time of 5 years (60 months) based on the latest patient record review (February 2022). The inclusion criteria were a primary diagnosis of sPTC, defined as a tumour ≤20 mm in size, and T1 stage with (N1) or without (N0) cervical lymph node metastases. The N1 group was further divided into N1a and N1b subgroups based on the location of metastases, with N1a classified as metastases in the central cervical lymph nodes (level VI) and N1b classified as metastases to the ipsilateral, bilateral, or contralateral lateral cervical lymph nodes (levels I-V). Patients with Nx lymph node status (where central lymph nodes could not be assessed or fewer than six central lymph nodes could be found) were not included. Patient inclusion was based on the 7^th^ edition of the tumour–node– metastasis (TNM) classification that was current during the study period [33].

Patients were subsequently subdivided into two groups based on whether they received intervention due to cancer recurrence (“recurrence group”) or not (“recurrence-free group”). Intervention-requiring cancer recurrence was defined as any type of clinical or radiological evidence of recurrence at least 3 months after the initial surgery, requiring any type of surgery for local and/or regional lymph node recurrence and/or RAI ablation, EBRT or other systemic treatment. All intervention-requiring recurrences were histologically confirmed by biopsy. Four patients in the cohort (two in the N1a group and two in the N1b group) had elevated thyroglobulin levels but no evidence of structural disease was seen during the follow-ups, and thus no extra treatment beyond the standard postoperative RAI was given. These four patients were included in the recurrence-free group.

**Fig. 1:**
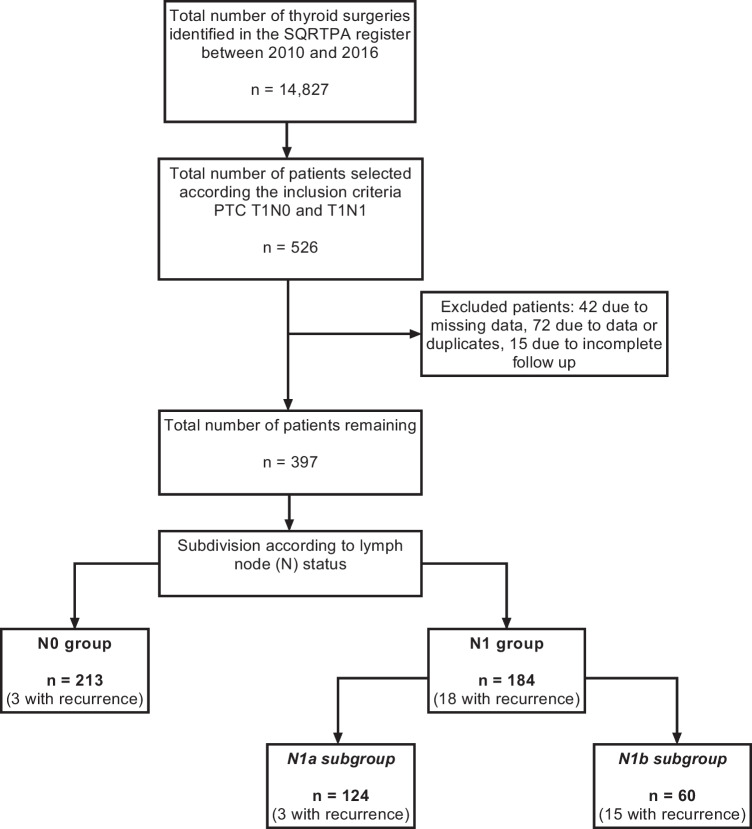
Patient selection

Exclusion criteria were patients that were alive but did not complete a minimum of 5 years (60 months) of follow-up time (*n* = 15), patients with insufficient medical record information or no access to patient medical records (*n* = 42). The study cohort was further checked for duplicates and any duplicate records were excluded (*n* = 72) (Fig. 1). One patient died from PTC before the 5-year minimum follow-up time (after 20 months) but was still included for further analysis. Tumours ≤20 mm formerly classified as T3 have been debated and re-classified in TNM 8th edition [33]. Thus, most of such tumours would be downstaged to T1 tumours according to size. As it was not possible to differentiate this in the register, these patients were excluded in order to have a pure and validated T1 cohort. No T4 tumours ≤20 mm were registered in the SQRTPA during the period. Only patients with M0 (no distant metastasis) status at the time of the diagnosis were included in this study.

### Ethical considerations

This study was approved by the Umeå Regional Ethics Committee, permit number 2021-01664, and was carried out in accordance with the EU’s General Data Protection Regulation (GDPR) rules.

### Statistics

Descriptive statistics were used to describe the basic characteristics of the three groups (N0, N1a, N1b) and recurrence vs. recurrence-free groups. One-way ANOVA and Pearson’s Chi-square test for categorical variables were used to assess the relationship between each group with the following potential predictors of recurrence: age, sex, tumour size, total number of metastatic LNs, multifocality, recurrence and primary RAI treatment. The Cox proportional hazard model was chosen for multivariable analysis to check for patient demographics and tumour variables.

A recurrence event was defined as described in the cohort selection section, above. The time to recurrence was quantified in months from day of primary surgery to time of the re-evaluation that confirmed the first PTC recurrence during follow-up. Disease-free survival in each group was estimated using the Kaplan–Meier curve, and the estimates among the groups were compared by the log-rank test. In all statistical analyses, a two-tailed p-value < 0.05 was considered statistically significant.

The statistical analyses and graphs were performed using SPSS version 27 (IBM Corporate, Armonk, NY) and GraphPad Prism version 9 (GraphPad Software, San Diego, CA).

## Results

The clinicopathological characteristics of the patients regarding nodal status (N0, N1a, N1b) are presented in Table 1. The median age of the patients in the N1b group was lower than the other groups (40.5 years; *p* = 0.002). The female/male ratio was higher in all groups (*p* = 0.003). The mean tumour size was smaller in both the N0 and N1b groups compared to N1a (9.3 mm and 9 mm compared to 11.8 mm, respectively; *p* = 0.001), and more tumours were T1a (1-10mm in size) in the N1b group (60%) compared with the N1a group (37.9%, *p* = 0.007). The mean number of metastatic LNs was significantly higher in the N1b group than the N1a group (6.64 and 3 respectively, *p* = 0.001) and the number of patients having five or more metastatic lymph nodes was significantly higher in the N1b group compared to N1a (41.7% vs. 12.9%, respectively; *p* = 0.001). Intervention-requiring cancer recurrence occurred more often in the N1b group (25%) compared to N1a (2.4%) and N0 groups (1.4%, *p* = 0.001). The overall intervention-requiring cancer recurrence rate was 5.3% (*n* = 21).

**Table 1:** Demographics and histopathology of the study population grouped by lymph node status (N0, N1a & N1b). One-way ANOVA test was used for comparison between continuous variables. Pearson’s Chi-square test was used for comparison between categorical variables

	N0	N1		*p*-value
		N1a	N1b	
Number of patients *n* (%)	213 (53.7%)	124 (31.2%)	60 (15.1%)	
Median age at diagnosis years, (min-max)	49 [16-83]	45 [12-83]	40.5 [17-85]	**0.002**
Age ≥ 45 years *n* (%)	127 (59.6%)	61 (49.2%)	21 (35%)	
Age < 45 years *n* (%)	86 (40.4%)	63 (50.8%)	39 (65%)	
Sex n (%)				**0.003**
Female	178 (83.6%)	100 (80.6%)	38 (63.3%)	
Male	35 (16.4%)	24 (19.4%)	22 (36.7%)	
Mean size of largest tumour in mm (min-max)	9.3 [1-20]	11.8 [1-20]	9 [1-20]	**0.001**
T1a, largest tumour size 1-10 mm *n* (%)	122 (57.3%)	47 (37.9%)	36 (60%)	**0.007**
T1b, largest tumour size 11-20 mm *n* (%)	91 (42.7%)	77 (62.1%)	24 (40%)	
Multifocality *n* (%)	65 (30.5%)	46 (37.1%)	28 (46.7%)	0.058
Mean number of metastatic LNs	-	3	6.6	**0.001**
Less than five metastatic LNs *n* (%)	-	108 (87.1%)	35 (58.3%)	**0.001**
Five or more metastatic LNs *n* (%)	-	16 (12.9%)	25 (41.7%)	
Primary postoperative RAI therapy *n* (%)	91 (42.7%)	120 (96.8%)	59 (98.3%)	**0.001**
Intervention-requiring tumour recurrence *n* (%)	3 (1.4%)	3 (2.4%)	15 (25%)	**0.001**
Recurrence type (n)*				
Local thyroid tissue	1	1	4	
Lymph node	2	2	10	
Thyroglobulin (Tg) / anti-Tg elevation	1	2	12	
Distant metastasis	0	0	3	
Treatment of recurrence (n)**				
Surgery	2	2	11	
Additional RAI	1	2	10	
External beam radiation therapy (EBRT)	0	0	3	
Systemic therapy	0	0	1	
Mean length of follow-up, months (min-max)	102.7 [60-145]	104.2 [61-145]	101.2 [20-145]	0.692

Statistically significant *p*-values are indicated with bold entries

* Some patients had multiple recurrences at the same time, and some had different types of recurrence more than one time

** Some patients received several types of treatments

The clinicopathological characteristics of the patients regarding intervention-requiring cancer recurrence (recurrence vs. recurrence-free groups) are presented in Table 2. The mean number of metastatic lymph nodes was higher in the intervention-requiring cancer recurrence group compared to the recurrence-free group (7 vs. 3.9; *p* = 0.006). The number of patients with N1b lymph node status was significantly higher in the intervention-requiring recurrence group compared to the recurrence-free group (71.4% vs. 12%, *p* = 0.001).

**Table 2:** Comparison between the intervention-requiring tumour recurrence group and recurrence-free group regarding risk factors. One-way ANOVA test was used for comparison between continuous variables. Pearson’s Chi-square test was used for comparison between categorical variables

	Intervention-requiring cancer recurrence	Recurrence-free	*p*-value
Number of patients *n* (%)	21 (5.3%)	376 (94.7%)	
Median age at diagnosis in years (min-max)	46 [26-85]	47 [12-85]	0.509
Age ≥ 45 years *n* (%)	11 (52.4%)	198 (52.7%)	
Age < 45 years *n* (%)	10 (47.6%)	178 (47.3%)	
Sex			0.401
Female *n* (%)	6 (28.6%)	301 (80.1%)	
Male *n* (%)	15 (71.4%)	75 (19.9%)	
Mean size of largest tumour in mm (min-max)	11.4 [1,5-20]	9.7 [1-20]	0.273
T1a, largest tumour size 1-10 mm *n* (%)	8 (38.1%)	197 (52.4%)	
T1b, largest tumour size 11-20 mm *n* (%)	13 (61.9%)	179 (47.6%)	
Multifocality *n* (%)	6 (28.6%)	133 (35.4%)	0.642
Mean number of metastatic LNs *n* (min-max)	7 [1-21]	3.9 [1-23]	**0.006**
Nodal status *n* (%)			**0.001**
N0	3 (14.3%)	210 (55.9%)	
N1a	3 (14.3%)	121 (32.1%)	
N1b	15 (71.4%)	45 (12%)	
Mean length of follow-up, months (min-max)	90.7 [20-120]	103.7 [60-145]	0.012

Statistically significant *p*-values are indicated with bold entries

**Fig. 2:**
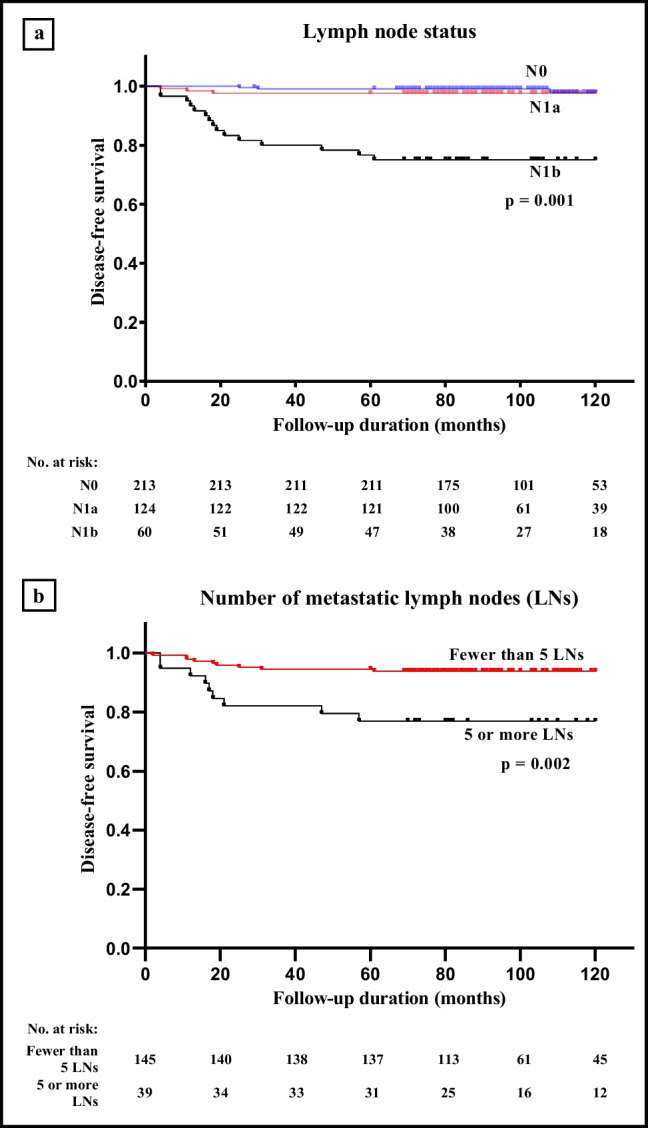
Kaplan–Meier curve and the log-rank test for disease-free survival of PTC patients regarding lymph node status (N0, N1a, N1b) (*p* = 0.001) (**a**) and the number of metastatic lymph nodes (LNs) (*p* = 0.002) (**b**)

The relationship of disease-free survival (DFS) between the two groups (intervention-requiring cancer recurrence vs. recurrence-free) is described in Fig. 2. The Kaplan–Meier curve and log-rank test for DFS demonstrate a significant difference between the two groups regarding LN status (N0, N1a and N1b, *p* = 0.001) in Fig. 2a, and between the number of metastatic lymph nodes with five LN as cut-off (*p* = 0.002) in Fig. 2b.

Using the Cox proportional hazard model for regression analysis to assess factors predictive of DFS, only LN status N1b compared to N1a and N0 (hazard ratio (HR) 19.93; 95% confidence interval (CI), 5.76 - 68.82, *p* = 0.001), and the number of metastatic LNs (five or more) (HR 3.98; 95% CI 1.58 - 10.03, *p* = 0.003) predicted decreased DFS as shown in Table 3.

**Table 3:** Risk factors for intervention-requiring tumour recurrence using Cox proportional hazard models

Variable	Hazard ratio	95% CI	*p*-value
LN status			
N0 group	1		
N1a group	1.73	0.35 - 8.56	0.501
N1b group	19.93	5.76 - 68.82	**0.001**
Age			
Age ≥ 45 years (*n*)	1		
Age < 45 years (*n*)	1.03	0.44 - 2.43	0.945
Sex			
Female	1		
Male	1.59	0.617 - 4.10	0.337
Largest tumour size in mm			
T1a, largest tumour size 1-10 mm	1		
T1b, largest tumour size 11-20 mm	1.77	0.74 - 4.28	0.201
Multifocality			
Unifocal	1		
Multifocal	0.74	0.288 - 1.91	0.538
Number of metastatic LNs			
Fewer than 5	1		
5 or more	3.98	1.58 - 10.03	**0.003**

Statistically significant *p*-values are indicated with bold entries

## Discussion

In this study, a cohort of 397 patients with sPTC (T1) was evaluated regarding intervention-requiring cancer recurrence during a follow-up period of at least 60 months. Basic clinicopathological characteristics regarding both the LN as well as the recurrence status were analysed. The recurrence rates in this study are similar to the ones described in the literature [9]. The overall recurrence rate was 5.3% (*n* = 21/397) when calculating for intervention-requiring cancer recurrences. The recurrence rate was significantly higher in the N1b group (25%) but mortality is still very low. Only one patient in the whole cohort of 397 died from thyroid cancer. The results support the hypothesis that recurrence rate, rather than mortality rate, is a preferable clinical measure when discussing sPTC. Given the difference regarding intervention-requiring cancer recurrence between N1b and N1a/N0, LN status plays an important role in individual risk stratification, and this emphasizes the need for thorough preoperative LN mapping even in sPTC [8]. Central lymph node metastasis (CLNM) has been shown to be the strongest risk factor for lateral lymph node metastasis. The sensitivity of the preoperative palpation or ultrasound for CLNM detection is low, especially when it comes to microscopic disease. This puts patients with PTC at risk of being under-staged and under-treated [34, 35]. One can argue that routine prophylactic central lymph node dissection is needed in order to obtain more appropriate risk stratification, but this leads to an increased risk for postoperative complications such as hypoparathyroidism and recurrent laryngeal nerve injuries, especially in low volume centres [35]. Furthermore, there are reports that postoperative RAI treatment has a negative impact on overall wellbeing and could possibly induce other malignancies [36]. More studies with large numbers of patients are warranted to find out whether our results imply an overtreatment of some sPTCs (N1a) when it comes to central lymph node surgery and postoperative RAI therapy.

Age is one of the most important prognostic factors for cancer-specific mortality in patients with PTC and it is currently evaluated together with other factors when assessing the risk for recurrences. The 8^th^ edition of the American Joint Committee on Cancer (AJCC), has raised the age cut-off from 45 to 55 years, thus reclassifying many patients <55 years with differentiated PTC and no evident distant metastasis (M0) as stage I [4]. As patients in this study were treated according to the 7^th^ edition of AJCC with an age cut-off of 45, this classification was kept. The median age in the N1b group (and N1 group in total) was lower than that of the N0 group (Table 1). However, the median age did not differ significantly between the groups when comparing recurrence and tended to be slightly lower in the intervention-requiring cancer recurrence group (Table 2). In both groups the median age was above the cut-off of 45 years.

The female/male ratio was significantly higher in all groups regarding LN status (Table 1), but no difference was observed when comparing recurrence status (Table 2) or DFS. Whether male sex is a predictive risk factor for recurrence has been controversial, but our results are in line with the latest data showing that female sex is a risk factor for PTC in general, but is not considered as a risk factor for recurrence in PTC or impact on the DFS [26, 28].

Tumour size ≥20mm, especially in combination with other risk factors such as BRAF and/or TERT mutations or ETE, is an established risk factor for LN metastasis and recurrence [22]. Well-differentiated PTMCs without worrisome features are considered as extremely low risk tumours and can be treated with lobectomy only, without the need for prophylactic LN dissection or RAI treatment. However, some of these tumours do recur with a rate ranging between 1% - 5% depending on multifocality. One study showed that 50% - 60% of patients with PTMCs have micrometastases that can be indolent and subclinical for several years [37]. Interestingly, we found that mean tumour size was significantly smaller in the N1b group compared to the N1a group. However, there were no significant differences in the recurrence groups or effect on the DFS, indicating that size alone cannot be considered a robust predictor for recurrence in sPTC.

Multifocality is considered a prognostic marker and risk factor for PTC recurrence, especially if more than two foci are present [22, 38, 39]. In our results, multifocality was not a risk factor for recurrence or decreased DFS.

These results show that LN status at presentation (N0, N1a or N1b) and clinical N1 status at diagnosis is correlated with risk for recurrence, a finding that is in line with previous publications [9, 22]. The number of metastatic lymph nodes is usually weighed together with the size of the metastasis and presence of ENE. According to the 2015 American Thyroid Association (ATA) guidelines, five or more metastatic LNs are considered as a risk factor for recurrence in patients with PTC [10, 19, 22, 23, 38]. In our cohort, analysis of the recurrence groups confirmed this (Tables 2 and 3, Fig. 2b). Regarding LN status as a separate risk factor, most of the patients in the N1a and N1b groups had fewer than five metastatic LNs (Table 1). However, when analysing the recurrence groups regarding LN status and number of metastatic LN nodes, our study results show that 60% (9/15) of the patients in the N1b group in the recurrence group had five or more metastatic LNs. In some cases of low-risk PTCs with tumour size up to 4 cm and without signs of LN metastasis or ETE, the latest ATA guidelines recommend hemithyroidectomy without the need of completion hemithyroidectomy or post operative RAI as sufficient treatment [38]. According to our results, this recommendation (and the active surveillance approach for PTMC) should be carefully considered as tumour size alone should not be used singularly for the preoperative surgical decision, rather in combination with other factors such as thorough preoperative LN status mapping and cytology with molecular analysis.

The strengths of this study are the accurate and reliable patient data based on thorough revision of medical and pathological reports in addition to the validated SQRTPA register data, as well as the follow-up time of at least 60 months (5 years) for all patients and up to 145 months (12 years) for some. The limitations of the study are the lack of molecular analyses of the tumours such as BRAF or TERT mutations, as these analyses were not routinely performed during the study period and not included in the Swedish national guidelines. Information on the size of the metastasized LNs or the ENE was not obtained, as this was lacking in many patients’ medical records. ETE of the tumour is an established risk factor for recurrence. However, as tumours ≤ 20mm with minimal ETE were classified as T3 during the study period, these could not be identified in the register and were therefore not included in the study.

## Conclusions

According to the present results, lateral lymph node metastasis (N1b) at the time of diagnosis and five or more metastatic LNs are the strongest predictors for cancer recurrence in patients with sPTC. Other factors cannot predict recurrence on their own, and should be used in combination with molecular testing to add strength to appropriate individual risk stratification. Small PTC should be considered as a heterogenous disease in which both treatment and follow-up should be individualized and tailored for each patient based on the clinicopathological features of the tumour and the presence of lymph node metastasis.

## List of abbreviations

TC: Thyroid cancer
PTC: Papillary thyroid cancer
sPTC: Small papillary thyroid cancer
PTMC: Papillary thyroid microcarcinoma
RAI: Radioactive iodine
TST: Thyroxin suppression therapy
EBRT: External beam radiation therapy
ETE: Extra thyroidal extension
ENE: Extra nodal extension
LN: Lymph node
SQRTPA: Scandinavian Quality Register for Thyroid, Parathyroid and Adrenal Surgery
DFS: Disease-free survival
CLNM: Central lymph node metastasis
AJCC: American Joint Committee on Cancer
